# Reuse of Uncured Carbon Fiber Prepreg Scrap via Calendering for Circular Composite Manufacturing

**DOI:** 10.3390/polym18151824

**Published:** 2026-07-25

**Authors:** Mónica Peñas-Caballero, Fernando Ramos Delgado, Víctor Gregorio, Eunice Cunha, María Ariño, Martín Bergaño Guzmán, Noelia Salmerón Pérez, Tamara Blanco Varela, Juan P. Fernández-Blázquez, Álvaro C. Casanova

**Affiliations:** 1 Foundation for the Research, Development and Application of Composite Materials (FIDAMC), Avenida Rita Levi Montalcini 29, Tecnogetafe, 28906 Getafe, Madrid, Spain; fernando.ramos@fidamc.es (F.R.D.); victor.gregorio@fidamc.es (V.G.); eunice.freitas@fidamc.es (E.C.); maria.arino-palacin@airbus.com (M.A.); alvaro.calero@fidamc.es (Á.C.C.); 2 IMDEA Materials Institute, C/Eric Kandel 2, 28906 Getafe, Madrid, Spain; martin.bergano@imdeamaterials.org; 3 AIRBUS Operations S.L., Paseo John Lennon s/n, 28906 Getafe, Madrid, Spain; noelia.salmeron-perez@airbus.com (N.S.P.); tamara.blanco@airbus.com (T.B.V.)

**Keywords:** uncured prepreg scrap, prepreg, carbon fiber, calendering, 2D X-Ray diffraction, fiber orientation

## Abstract

This technical study presents calendering as an innovative and continuous industrial solution for the reuse of uncured carbon fiber prepreg scraps, which are typically discarded in large quantities in the aerospace sector. By applying heat and pressure through rollers, the authors successfully transform residual fragments into functional sheets, promoting a circular economy that reduces environmental impact and raw material costs. Although the process induces a slight fiber misalignment, which decreases longitudinal strength, the results reveal significant improvements in transverse properties and increased material isotropy. Finally, the research validates the feasibility of this technique by fabricating a curved structural demonstrator, proving that these recycled composites maintain high stiffness and suitable thermal stability for secondary aeronautical applications.

## 1. Introduction

Industries such as aerospace, automotive, wind energy, and sports equipment have raised increasing concerns regarding the sustainability of composite manufacturing processes [1,2]. While CFRP materials offer exceptional mechanical performance, low weight, and corrosion resistance, their production is energy-intensive and relies on high-cost raw materials obtained from fossil feedstocks.

Considering these sustainability concerns, recent life cycle assessment (LCA) studies emphasize that the environmental benefits of CFRPs are highly dependent on their operational lifespan. For instance, while their production footprint is high, CFRPs could become more ecologically efficient than traditional aluminum alloys after a break-even distance for an aircraft of approximately 300,000 km in-service, provided that effective end-of-life and recycling strategies are integrated [1].

Complementing these end-to-end life cycle considerations, the scale of current industrial demand further intensifies the need for resource efficiency. Global consumption of carbon fiber is estimated at approximately 110,000 tons per year, with a substantial portion dedicated to the aerospace sector. In modern aircraft models, such as the Airbus A350, composites now account for over 50% of the total structural weight [2,3,4]. However, the transition to these new materials introduces significant manufacturing challenges, particularly regarding material waste. The industry-standard ‘buy-to-fly’ ratio between the weight of the raw material purchased to manufacture a part and the weight of the final part installed in the aircraft is approximately 1.3:1 for automatic lay-up processes, meaning that roughly 30% of the material is lost during processes such as ply cutting, contouring, or manufacturing errors [5]. Critically, approximately 80% of this waste consists of uncured prepreg scrap, which is typically discarded without further use, presenting a significant opportunity for repurposing in secondary structural applications [6,7,8].

One promising pathway towards sustainability is the reuse of CFRP prepreg materials, particularly in the form of manufacturing scraps. During cutting, trimming, and lay-up operations, significant amounts of uncured prepreg waste, usually made of several layers, are generated. These scraps often retain high-quality carbon fibers and partially reactive resin systems, thereby preserving their intrinsic material value. Instead of being discarded or downgraded through conventional recycling methods, these prepreg leftovers could potentially be repurposed into new composite products. Direct reuse strategies preserve fiber architecture and mechanical properties whilst reducing both raw material consumption and production costs.

Several recycling methods have been widely developed for CFRP waste, including mechanical grinding, thermal processes such as pyrolysis, and chemical solvolysis [9,10,11,12]. Mechanical recycling methodologies, which allow recovery of both resin and fiber, unfortunately, tend to result in significantly degraded material properties due to grinding. Other techniques, such as pyrolysis and solvolysis, primarily recover fiber but often entail drawbacks, including high energy consumption, complex processing steps, and the degradation of fiber properties and resin systems. Consequently, these recovered short fibers typically need to be reprocessed into secondary products, such as non-woven mats or short-fiber composites, thus restricting their use to low-performance applications [13,14]. As a result, these traditional recycling routes tend to struggle to deliver both environmental and economic efficiency, particularly for high-value prepreg waste.

In contrast, uncured prepreg scrap recycling through direct consolidation techniques offers a more sustainable and attractive industrial alternative. Souza et al. [15] demonstrated that randomly distributed ply-cutting scraps can produce laminates with a 34% increase in interlaminar shear strength (ILSS) and a specific strength exceeding that of aircraft aluminum, particularly for tooling applications. This approach was further refined by Wu et al. [16] who investigated the compression molding of scrap ‘chips’ or strands. Their work identified that by controlling process parameters, such as chip geometry and cure cycles, it is possible to minimize porosity and optimize microstructural quality, proving the technical viability of converting random waste into high-quality parts. Conversely, to address structural load-bearing requirements, Bettini et al. proposed a more controlled architecture using ‘patched prepreg sheets’. By assembling scraps into regular, engineered patterns rather than random distributions, they successfully recovered up to 90% of the original material stiffness [17].

While studies by Souza et al., Wu et al., and Bettini et al. have successfully demonstrated the mechanical feasibility of reusing prepreg scraps through discontinuous processing methods such as compression molding, a significant gap remains regarding industrial scalability. To address this, this study introduces calendering as a high-throughput, continuous alternative. Unlike static molding, calendering applies controlled heat and pressure through rotating rollers to consolidate fragmented uncured scraps into isotropic-like, ready-to-use intermediate sheets. As highlighted by Milite et al. [18], a critical challenge in managing uncured prepreg waste is the development of efficient, scalable cutting technologies that maximize patch nesting efficiency and minimize irregular patches that may compromise ultimate mechanical properties. By shifting from manual or batch-based assembly to a continuous calendering process, this study aims to bridge the gap between laboratory-scale recycling and large-scale aeronautical manufacturing.

This study explores the feasibility of reusing uncured carbon fiber prepreg multilayered scraps through calendering consolidation. The influence of processing parameters on material quality, bonding mechanisms, and mechanical performance is systematically analyzed. The results aim to demonstrate the potential of calendering as a scalable, low-waste, and cost-effective solution for the reutilization of uncured prepreg scrap. Ultimately, this approach contributes to the development of more sustainable composite manufacturing systems by reducing landfill disposal, lowering carbon emissions, and promoting circular material flow within the CFRP industry.

## 2. Materials and Methods

### 2.1. Materials

The material used in this study is a HexPly^®^ 8552/AS4 carbon fiber prepreg supplied by Hexcel Composites (Stamford, CT, USA) for aeronautical applications. This material provides high mechanical strength combined with low weight. It exhibits a fiber volume fraction (Vf) of 59%, a gravimetric density of 1.59 g/cm^3^, and a ply thickness of 0.184 mm. The prepreg service temperature range is −55 °C to 95 °C and can be cured through two standard thermal cycles. It is stored frozen at −18 °C and has a shelf life of 1 year.

### 2.2. Scrap Preparation

The uncured prepreg scrap layouts were simulated and prepared following a symmetric quasi-isotropic laminate configuration, specifically [−45/+45/0_3_/90/0_3_/+45/−45]. This represents a typical aeronautical stacking sequence and ensures a balanced distribution of fiber orientations. Individual plies were manually cut and stacked according to the predefined lay-up sequence at room temperature under controlled laboratory conditions. The resulting stack reached a total nominal thickness of 2.32 mm. The laminate was compacted to ensure adequate ply adhesion while avoiding any premature resin flow or fiber distortion. The uncured laminates were cut into strips with nominal dimensions of 600 × 35 mm for subsequent calendering.

### 2.3. Scrap Reprocessing Procedure

**Figure 1:**
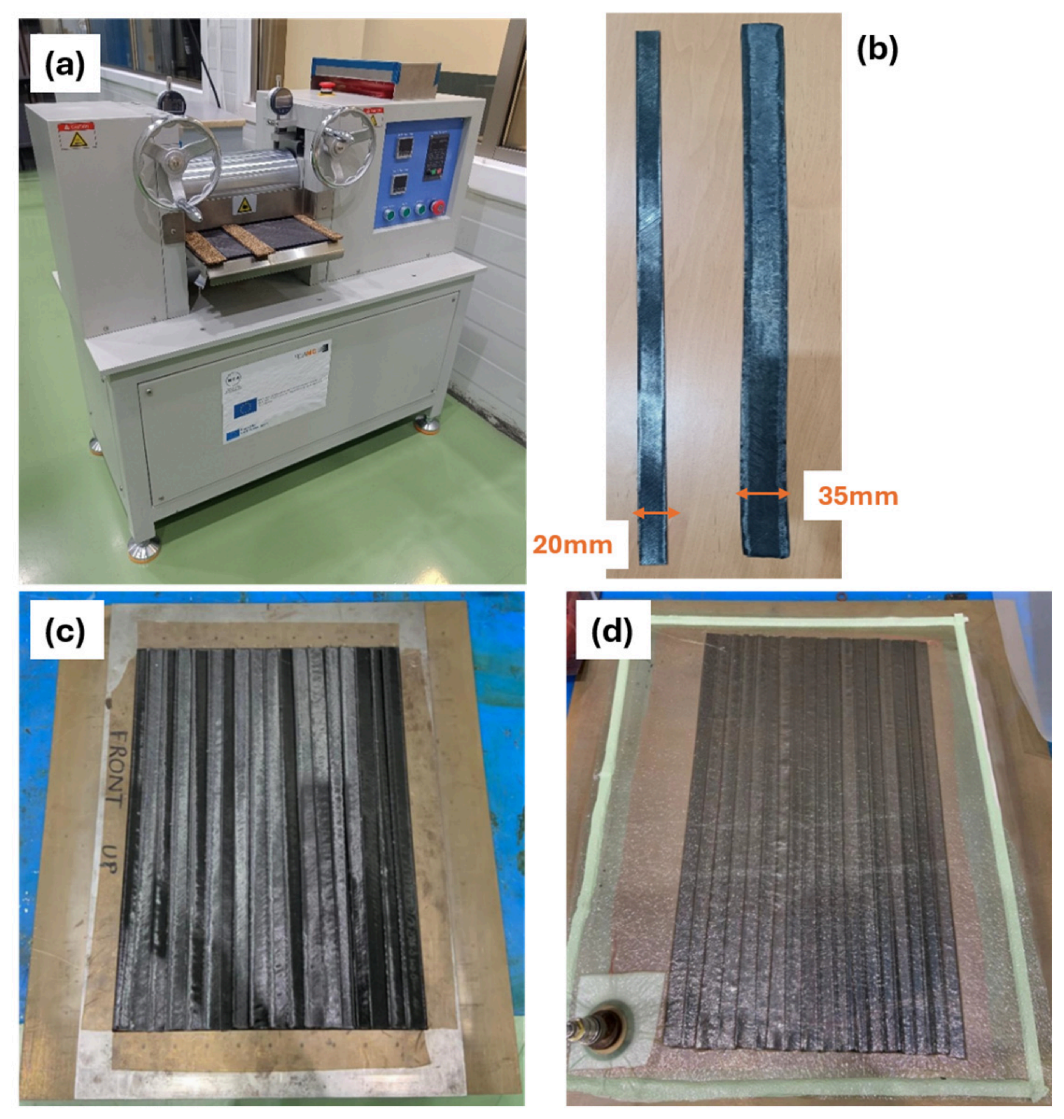
Calendering system and assembly process for prepreg scrap reuse. (**a**) TOB-DR-H150-200 roll heat press used for continuous consolidation. (**b**) Comparison of prepreg strips before and after calendering; the increase in width from 20 to 35 mm demonstrates material redistribution under heat and pressure. (**c**) Manual staggered placement of strips and (**d**) consolidation using a corrugated bag to ensure interlaminar nesting and void reduction prior to final curing.

To reprocess the pre-impregnated scraps, each strip is calendered using a TOB-DR-H150-200 roll heat press (TOB New Energy Co., Ltd., Xiamen, China) (Figure 1a) operating at a controlled temperature of 50–60 °C and a speed of 8.5 m·min^−1^. This procedure had been previously optimized, as the thickness reduction must be performed progressively with temperature. The roller gap decreased by 0.2 mm per pass until the target thickness was reached. A higher gap reduction in a single pass caused the fibers to cluster, resulting in a tangled, non-uniform structure.

During the calendering process, the prepreg initial thickness of 2.32 mm is reduced to 1.2 ± 0.2 mm, and the width is increased from 20 to 35 mm due to material redistribution (Figure 1b). The rollers were maintained at a constant temperature of 50–60 °C to facilitate material deformation. Before calendering, the strips were preheated with a hot-air dryer to reduce stiffness and prevent fiber recoil. Additionally, a slight tensile force was applied at both the inlet and outlet of the calender to maintain fiber alignment and ensure uniform thickness reduction.

### 2.4. Manufacturing of Test Panels

The panels were manufactured using a Marzola Hot Plate (Marzola Prensa Hidráulica, S.L., Logroño, La Rioja, Spain). Before fabricating the test panels, the scraps were arranged with a slight overlap of approximately 2–3 mm along the longitudinal edges of the strips to enhance adhesion between them. Once positioned, the material was compacted using a corrugated bag while applying heat to further improve bonding, as shown in Figure 1c,d.

The individual sheets were stacked to achieve the desired thickness, as specified by the test requirements. The panels were cured according to the material supplier’s instructions. Table 1 presents the manufactured panels, including layer orientations and target thicknesses for each configuration.

Reference laminates were also manufactured with a thickness of 2 mm for the experimental program, using the same virgin material and stacking sequence as the scraps: [−45/+45/0_3_/90/0_3_/+45/−45].

### 2.5. Demonstrator Fabrication

The demonstrator was manufactured using leading-edge panels (or skins) from the rudder of aircraft such as the A320 or A350. These components are curved, unstiffened structures classified as Category D (non-critical), where the design prioritizes stiffness to prevent deformation during flight rather than damage tolerance.

A custom mold was designed for hot press processing. The cavity dimensions are 1000.056 mm × 232.387 mm, with a curvature radius of 163.422 mm and general tolerances of ±0.2 mm. Calendered scraps were cut to a length of 200 mm and assembled in an overlapping configuration with a transverse offset of 2–5 mm to mitigate post-calendering longitudinal curvature and prevent interlaminar gaps. The preform dimensions were fixed at 900 mm to provide sufficient clearance for volumetric expansion and mold-filling during compression. This laminated structure consisted of three resin-rich layers, which served both as adhesives to stabilize the layers and as secondary matrices to fill interstitial voids.

The mold was initially closed under no pressure and heated to 70 °C; at this point, the pressure was increased incrementally to 8 bar. The system was then heated to a target temperature of 180 °C (platen temperature: 200 °C) and maintained for 120 min, with pressure continuously readjusted to compensate for the reduction in resin viscosity. Finally, a 60 min cooling phase was conducted under a constant 8 bar before demolding at ambient temperature.

### 2.6. Characterization Techniques

Each composite panel underwent a comprehensive physicochemical analysis to be compared against the virgin laminate properties. Fiber content was quantified by acid digestion in accordance with ISO 1183-1. The glass transition temperature (Tg) was determined using a Dynamic Mechanical Analysis (DMA) following AITM 1-0003. The analysis was performed using TA Instruments Q800 DMA (TA Instruments, New Castle, DE, USA) equipped with a single cantilever clamping geometry. Macroscopic and microscopic examinations were also performed, enabling the identification of internal defects and the assessment of local variations in resin content, under a Nikon Eclipse LV150 (Nikon Corporation, Tokyo, Japan) optical microscope.

Tensile tests were performed parallel and perpendicular to the fibers (EN 2561; EN 2597) using MTS Model 370.10 Universal Testing Machine (MTS Systems Corp., Eden Prairie, MN, USA), FLFMTS. Compression tests were conducted in accordance with EN 2580B, using type B1 specimens to determine tensile strength and type B2 specimens to determine tensile modulus. An MTS Criterion (Model 45) (MTS Systems Corp., Eden Prairie, MN, USA) universal testing machine was used. Additionally, the apparent interlaminar shear strength (ILSS) was measured in accordance with EN 2563 in a Zwick universal testing machine, FL-ZWICK (ZwickRoell, Ulm, Germany).

The fiber orientation was studied by wide-angle X-ray scattering (WAXS) using SAXSpoint 5.0 (Anton Paar GmbH, Graz, Austria) equipment. Measurements were performed exclusively in the wide-angle regime to analyze fiber orientation. The system was equipped with a CuKα microfocus source (λ = 1.5418 Å) and a two-dimensional Pilatus 1M (Dectris) detector (DECTRIS Ltd., Baden-Daettwil, Switzerland), which enabled analysis of fiber preferred orientation. 2D patterns were converted into 1D intensity profiles using the SAXS analysis software (version 2.56, Anton Paar GmbH, Graz, Austria).

## 3. Results and Discussion

### 3.1. Fiber, Resin, and Void Content

To evaluate the reproducibility of the calendering and hot-pressing processes, the physicochemical properties of the prepared laminates were examined.

Table 2 summarizes the measured density, fiber, resin, and void contents of the fabricated panels. The Vf ranged from 55.4% to 58.2% and showed a good correlation with the reference sample (58.6%). Although these fluctuations might appear relevant at first glance, the variation between different panels (approx. 1.5%) is lower than the experimental error observed within single samples (up to 2.0%). This indicates that the process is remarkably consistent, and the observed deviations are likely due to localized fiber overlaps and resin-deficient regions inherent to the manual lay-up and calendering of scraps, rather than fundamental differences between panels.

A similar trend occurs with the void content Vo, where values remain low (between −0.4 and 0.9%). Despite these fluctuations, the obtained values are consistent with the reference sample and fall within a reasonable range compared to those reported in the literature for high-performance composites [19,20]. Although the calendering process can slightly increase the void content due to the creation of dry areas during processing, as seen in P2 and P3 (0.9%), the results for P4 (0.1%) demonstrate that the process can achieve even higher consolidation than the reference. These results suggest that further investigation into the number of overlaps per laminate, ply count, and specific processing parameters will be key to identifying optimal settings to minimize porosity and maximize structural integrity.

### 3.2. Thermal Properties

**Figure 2:**
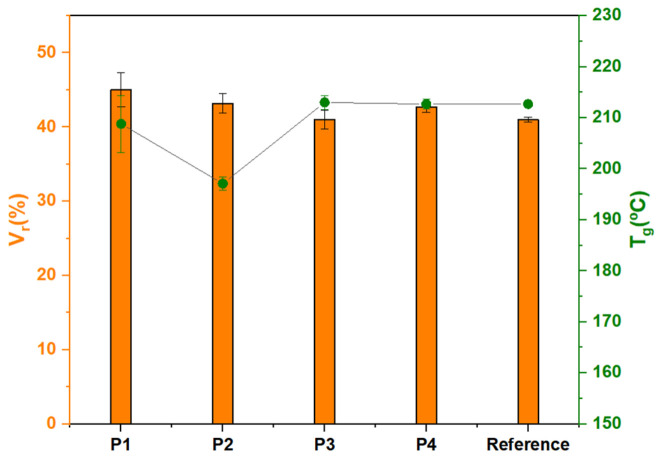
Thermal stability analysis. Comparative graph of Vr (left y-axis) and glass transition temperature (right y-axis).

Figure 2 compares the resin content (Vr) obtained by acid digestion to the glass transition temperature (Tg) values, estimated as the onset point of the storage modulus transition during DMA testing.

In this study, the P2 calendered sample shows slightly lower Tg value compared to the reference, while the other calendered samples (P1, P3 and P4) show a Tg within the typical DMA scatter. Nevertheless, these temperatures remain high across all samples thermal properties, and resin content was not altered significantly, which remains almost constant throughout the different tests.

### 3.3. Mechanical Properties

#### 3.3.1. Tensile Test

Tensile tests at 0° and 90° orientations were performed on the calendered and reference specimens. Table 3 presents the elastic modulus (E_T_), which was calculated over the most linear portion of each stress–strain curve, and the tensile strength (σ_T_).

The mechanical properties of the calendered samples in the fiber direction showed a significant reduction compared to the reference laminate. Calendered samples exhibited a 70% reduction in σ_T_, whereas E_T_ decreased by approximately 5%. This marginal variation in the elastic modulus suggests that the calendered material retains a stiffness comparable to the non-calendered material in the longitudinal direction, while the drastic drop in tensile strength indicates premature failure. Local random orientation and calendering-induced packing defects affect strength without significantly altering modulus, which depends more on the overall fiber volumetric fraction [21,22]. Furthermore, the high coefficient of variation reflects significant heterogeneity in fiber orientation within the calendered laminate.

This reduction in longitudinal tensile properties compared with the reference sample aligns with trends reported in the literature for recycled composites. For instance, Souza et al. (2019) [15] reported a 13% decrease in tensile strength using uncured prepreg scraps, while Bettini et al. and Wu et al. [16,17] observed reductions of 50% and 50–60%, respectively. In more extreme cases, Aravindan et al. [23] reported losses of up to 85.9% using waste carbon fibers. Overall, while the calendered material in this study exhibits a greater strength reduction than some previous published approaches, its ability to largely preserve stiffness remains a distinguishing characteristic.

In contrast to the longitudinal results, the tensile tests at 90° (Table 3) demonstrate significantly higher homogeneity, with coefficients of variation remaining below 5%. Also, the calendered samples exhibit enhanced transverse properties compared to the reference laminate. This improvement is primarily attributed to the calendering process, which partially randomizes the fibers. As fibers shift from their original longitudinal alignment toward a more transverse orientation, they provide additional reinforcement in the 90° direction.

This phenomenon is consistent with the findings of Feraboli et al., who demonstrated that the use of randomized prepreg scrap (chips) produces a quasi-isotropic material in which mechanical properties, particularly stiffness, are balanced across multiple orientations, effectively neutralizing the original anisotropic bias of the uncured waste [24]. Consequently, while this reorientation leads to a decline in fiber-parallel properties, it effectively improves the material performance in the perpendicular direction. Longana et al. suggest that the recycling process induces a slight fiber misalignment or ‘fanning out’ effect. These misaligned fibers act as transversal reinforcement, enhancing the matrix-dominated strength at 90° by providing mechanical bridging that is absent in a perfectly aligned unidirectional laminate [25].

**Figure 3:**
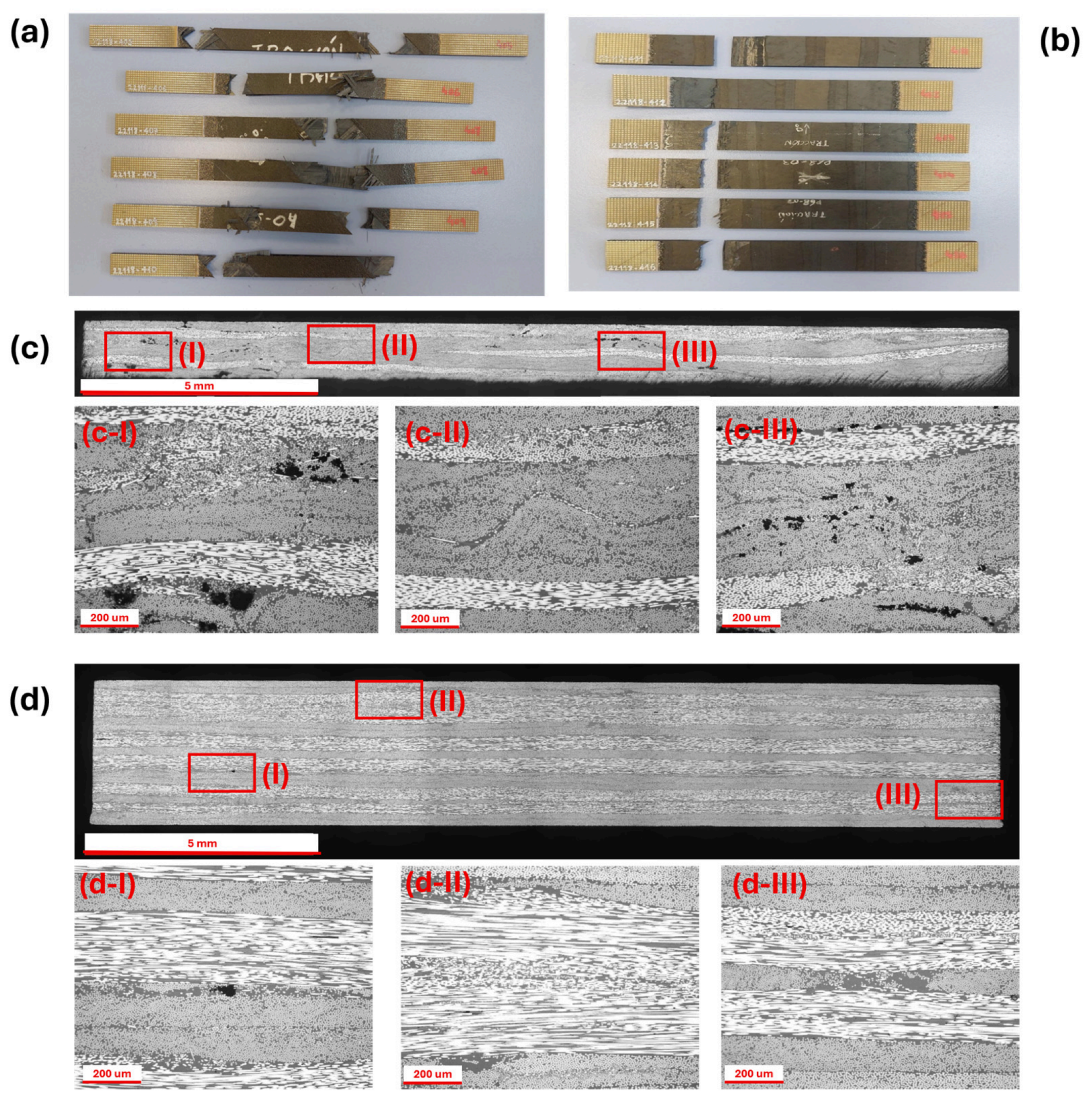
Failure mode characterization and microstructural defects. Visual evidence of gradual failure in (**a**) 0° specimens versus interface-driven failure in (**b**) 90° specimens. (**c**,**d**) Micrographs of specimen (**a**) and specimen (**b**); (**I**–**III**) and higher-magnification views of (**c**,**d**).

The observed failure mechanisms further clarify the material’s mechanical response. Regarding the longitudinal tests (0°), most specimens failed within the free length, considered a valid failure mode. However, two specimens exhibited mixed failure, with crack initiation in both the central section and at the tab ends. This behavior could be attributed to stress concentrations induced by material heterogeneity, as evidenced by the high coefficients of variation previously discussed. As shown in Figure 3a, failure in the calendered material is gradual, progressing through longitudinal cracking and delamination. This progressive failure is attributed to discontinuities at the interfaces between recycled scrap.

In contrast, as illustrated in Figure 3b for the 90° tests, failure initiated and propagated specifically along the bonding regions between adjacent strips. This phenomenon is clearly visible on the specimen surface, where the joining interfaces between the scraps correspond to the observed fracture path. This suggests that while the 90° properties are enhanced by fiber reorientation, the overall strength remains limited by the interlaminar or inter-strip bonding quality.

A microscopic evaluation was conducted to examine potential defects that may contribute to progressive failure of the material. Figure 3c,d presents representative areas of the specimens and evidence the fiber misalignment within plies and also between them. Micrographs (c–I) and (d–I) reveal the presence of some voids, which act as stress concentrators and are expected to initiate premature failure. These defects highlight the relevance of processing quality, proving that small voids can compromise the mechanical performance and durability of the composite.

#### 3.3.2. Compression Test

Compression tests, parallel and perpendicular to the fiber direction, were conducted in accordance with the relevant standards; the compressive strength (σ_C_) and modulus (E_C_) are summarized in Table 4. These outcomes follow a trend like that observed in the tensile tests. Specifically, the σ and E values are lower in the calendered samples and exhibit high variability across the different specimens. σ_C_ is reduced from 1531 MPa in the reference sample to 502 MPa upon calendering, with a highly significant scatter. E_C_ value for the calendered samples is around 60 GPa, which is 50% lower than the 125 GPa value measured for the reference. This behavior is fundamentally exacerbated by the calendering process; although effective for sheet forming, the mechanical pressure and material flow during calendering inevitably induce a loss of fiber orientation along the critical 0° direction. As a result, the material exhibits lower stiffness and reduced structural stability, as the load can no longer be efficiently transferred along the fibers’ longitudinal axis, a phenomenon that aligns with the results observed in the parallel tensile tests [26].

For the compression test at 90°, the results for the calendered material show no significant decrease, as was also observed in the tensile tests at 90°. In this case, the presence of randomly oriented fibers leads to improved properties, particularly the modulus, which increases the module by 280%, indicating greater stiffness of the samples in this direction. This enhancement can be attributed to fibers oriented at angles other than 90°, resulting from the calendering process.

**Figure 4:**
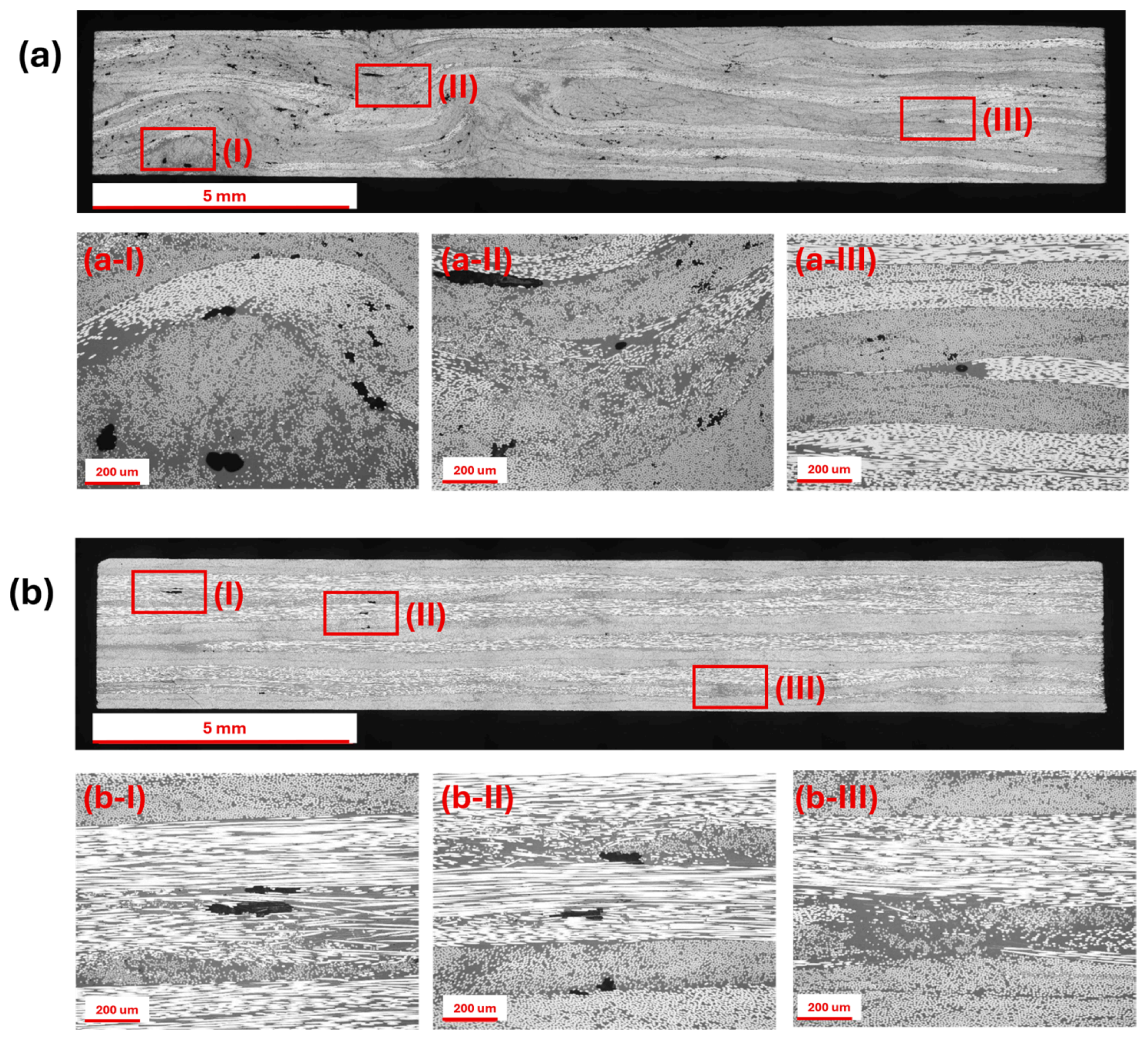
Microstructural assessment of consolidation quality. Cross-sectional views of (**a**) 0° and (**b**) 90° specimen 3.3.3 Comparative compression and tensile test; (**I**–**III**) and higher-magnification views of (**a**,**b**).

The microstructural quality of the demonstrator was validated through cross-sectional micrographs of the specimens used for mechanical testing, as shown in Figure 4. Figure 4a corresponds to the region prepared for 0° testing, where the analysis reveals high fiber misalignment, good consolidation, and good resin distribution. Although detailed observations in regions (a-I) and (a-II) indicate the presence of small, localized voids, which may act as stress concentrators and contribute, together with fiber misalignment, to the variability in longitudinal properties, their negligible size ensures that structural integrity is maintained. In contrast, Figure 4b displays the specimen used for 90° testing, where region (b-III) shows a completely defect-free interface with optimal interlaminar adhesion and fiber wetting. The absence of significant macro-defects or extensive dry zones in both orientations confirms the effectiveness of the manufacturing process and justifies the consistency observed in the 90° elastic modulus measurements.

**Figure 5:**
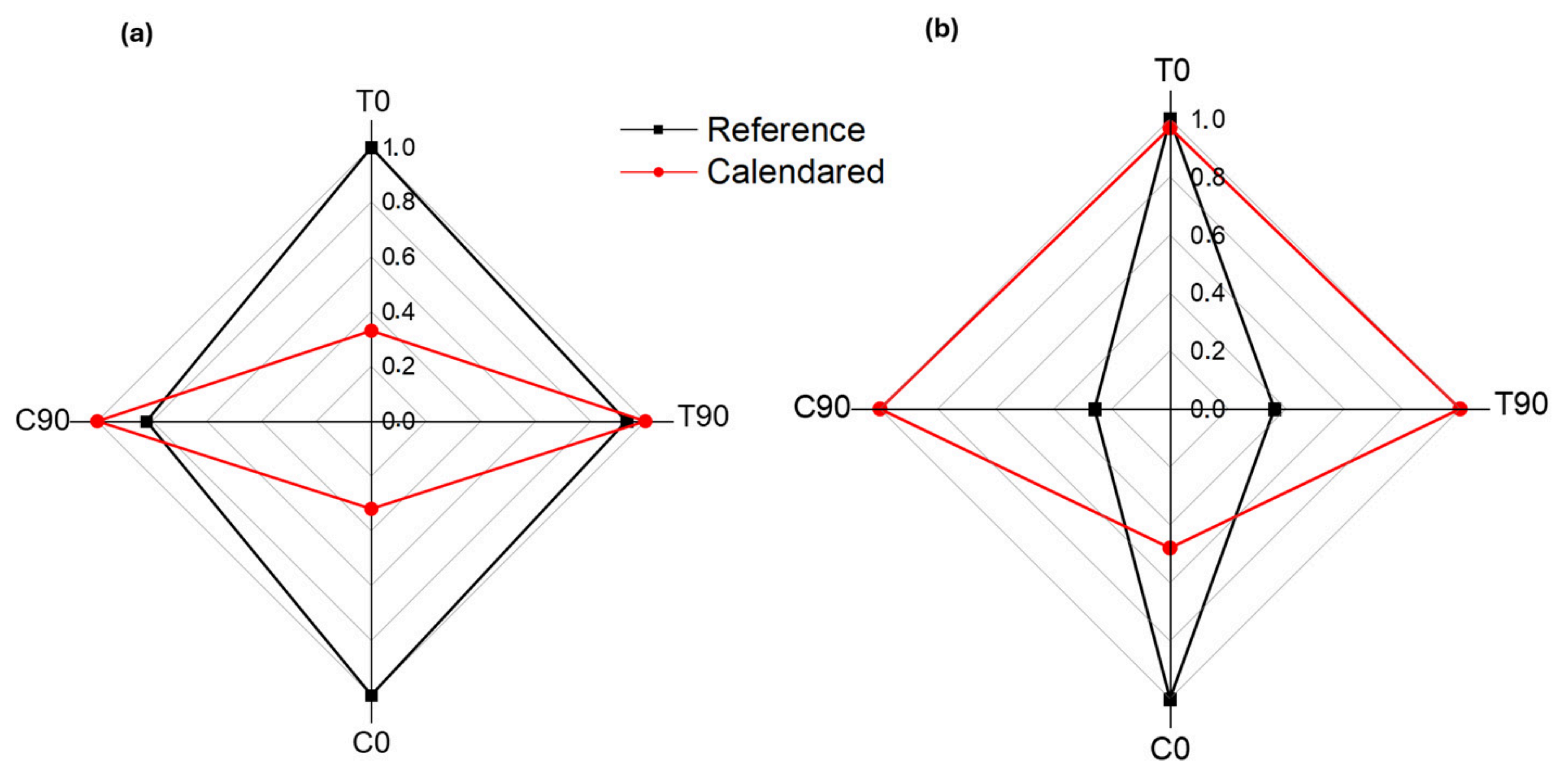
Comparative mechanical performance (Radar Charts) for (**a**) strength and (**b**) modulus across the four main testing conditions: Tensile 0° (T0), Tensile 90° (T90), Compression 0° (C0), and Compression 90° (C90).

The mechanical performance of the manufactured demonstrator was normalized against the reference material to evaluate the impact of the processing stages, specifically the calendering process, as shown in Figure 5.

Regarding mechanical strength, the reference material (black line) exhibits a highly anisotropic profile, with its maximum performance concentrated in the longitudinal axis (T0 and C0). In contrast, the calendered demonstrator (red line) shows a significant reduction in longitudinal strength, retaining only approximately 33% of the reference values. However, a notable enhancement is observed in the transverse direction (90°), particularly in compression (C90), where the strength exceeds the reference. This shift indicates a localized isotropization of the material’s resistance, likely due to the reorganization and fragmentation of the fiber architecture previously discussed.

The elastic modulus distribution (Figure 5b) reveals a different trend. While the longitudinal stiffness (T0) of the calendered sample remains comparable to the reference, the transverse stiffness (T90 and C90) shows a substantial increase. This “widening” of the radar plot in the horizontal axis demonstrates that the processing significantly stiffens the material in the 90° direction. The combination of maintained longitudinal modulus and enhanced transverse stiffness results in a more balanced structural response, albeit with the trade-off in strength in the 0° orientation.

#### 3.3.3. Interlaminar Shear Strength (ILSS)

The ILSS test evaluates the influence of scraps at the fiber/matrix interface, which is dominated by both the polymeric matrix and the fiber/matrix interface. The test was not conducted according to the previously described standard, as both the laminate and the thickness fell outside the standard specifications. Therefore, it was decided to study a reference specimen with the same dimensions for comparison purposes. Table 5 shows the calculated interlaminar shear strength and peak load values for the calendered specimen.

Table 5 shows a comparative study of the ILSS, which exhibits a marginal reduction of 4.4% (from 60 MPa to 58.0 MPa) when calendered material is used. This slight decrease is due to the discontinuous nature of the scrap-based laminate, where the interfaces between fragments act as preferential sites for shear crack initiation.

Conversely, the peak load increased by 20.3% (from 1735 ± 224 N for the reference to 2087 ± 273 N for the scrap-based specimen). These findings suggest that, while the intrinsic interlaminar strength is slightly compromised by the recycling process, this significant rise in load-bearing capacity can be attributed to local variations in specimen geometry, such as increased thickness (from 2.1 of referenced to 2.6 mm for the calendered sample) or localized resin enrichment at the fragment overlaps, which allow the material to support higher absolute loads before critical failure. These findings suggest that while the intrinsic interlaminar strength is slightly compromised by the recycling process, the macroscopic load response is enhanced by the structural heterogeneity and the redistribution of stresses through the randomized fiber architecture, a phenomenon that aligns with microstructural effects reported by Souza et al. [15].

**Figure 6:**
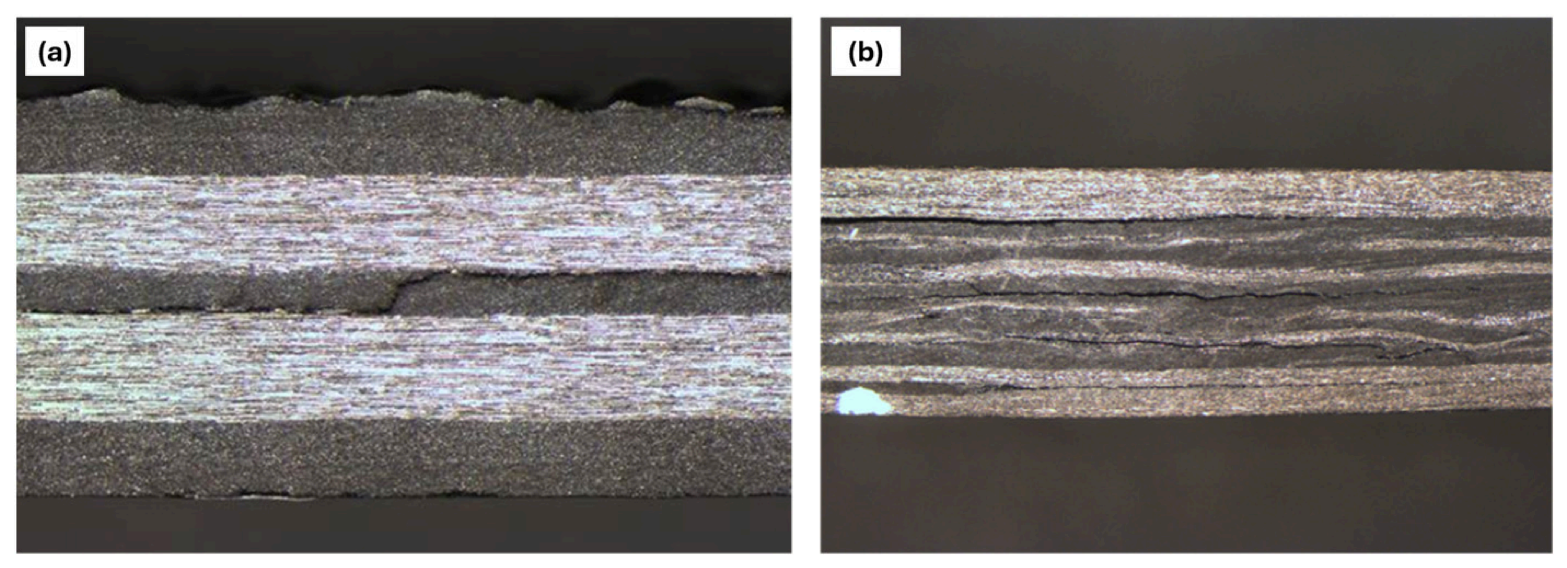
Visual appearance of the cross-section of the tested specimens for (**a**) reference and (**b**) calendered sample.

Figure 6 illustrates the specimens after mechanical testing, highlighting the crack propagation between laminates. For the reference sample, a single central crack is evident, while the calendered sample exhibits multiple cracks. This behavior can be linked to the random orientation of some carbon fibers within the calendered material. Such misalignment can impede the direct path of crack propagation, effectively delay failure, and enhance interlaminar shear strength by promoting a more uniform distribution of stress across the layers. This phenomenon was previously reported in a patch material laminate, where the crack initiates on the outer side of the sample (the one in tension during bending), breaks through five layers before reaching a new discontinuity, from which the delamination is triggered [27].

### 3.4. 2D WAXS Analysis of Fiber Orientation

**Figure 7:**
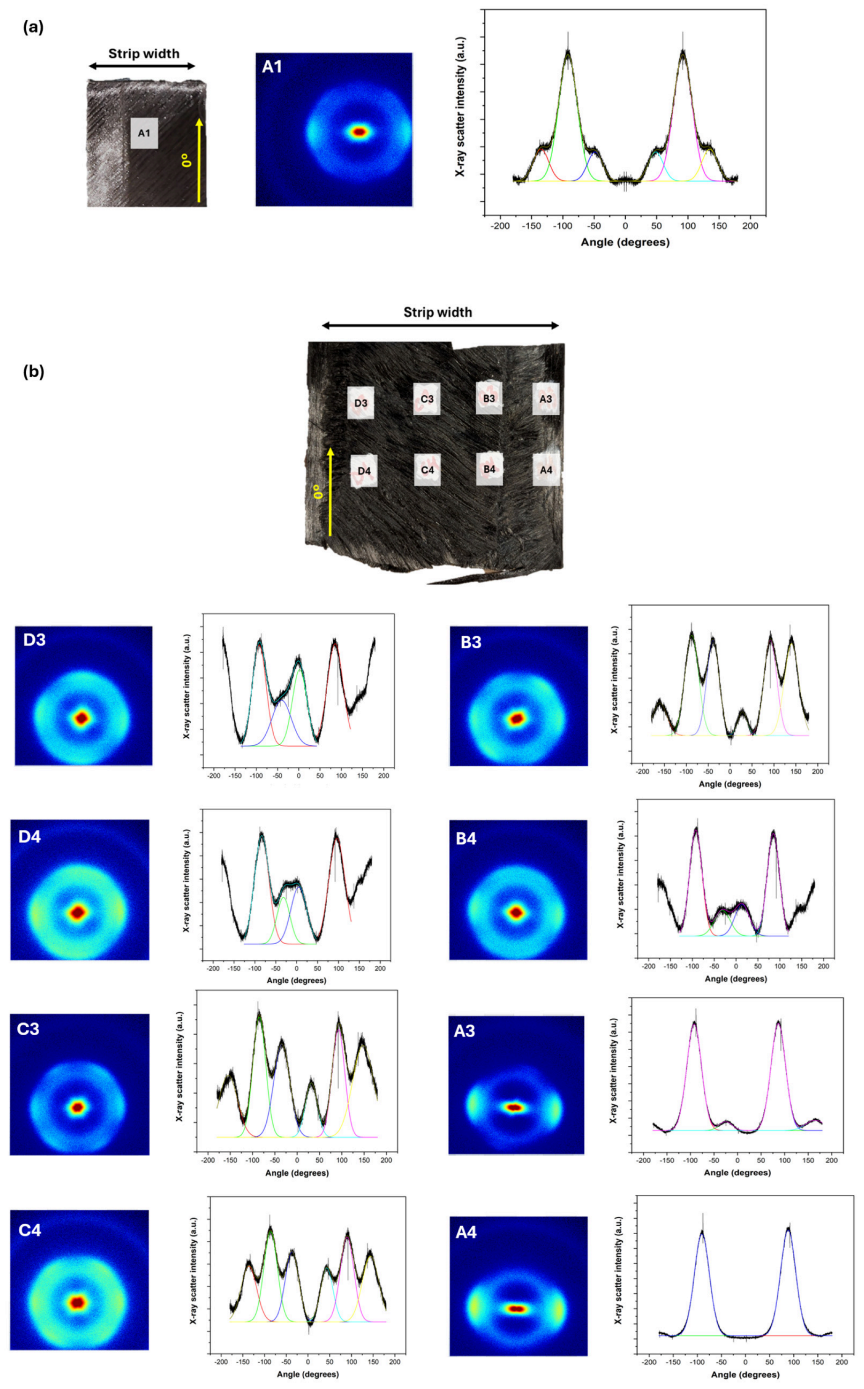
2D wide-angle X-ray scattering (WAXS) patterns. Diffraction patterns of (**a**) virgin and (**b**) calendered scraps. The broadening and intensity shift in the diffraction lobes provide direct experimental evidence of the loss of preferential fiber alignment (isotropization) during processing.

Figure 7 presents the two-dimensional WAXS patterns obtained from the virgin and calendered uncured prepreg scraps, along with the corresponding angular integration profile.

In all cases, the patterns exhibit a diffuse scattering halo, characteristic of a weakly ordered or amorphous structure. The intense central spot corresponds to the direct beam (the beamstop region, though this equipment allows us to work without a beamstop) and is unrelated to the material’s structural features. In addition, in some cases, well-defined intensity lobes are observed in all samples and are associated with the preferential orientation of the carbon fibers within each ply. These lobes arise mainly from the diffraction of the graphitic (002) planes from carbon fibers, which are oriented perpendicular to the fiber axis. Consequently, fibers aligned along the 0° prepreg direction give rise to maximum intensity in the equatorial plane of the 2D WAXS pattern (i.e., rotated by 90° with respect to the fiber direction), whereas fibers oriented at 90° are reflected in the meridional plane. Angular integration was performed for each of these 2D patterns to evaluate the relative contributions of the different carbon fiber orientation planes.

To quantitatively evaluate the preferential fiber alignments and the degree of disorientation induced by the calendering process, the profiles obtained from the azimuthal integration of the 2D WAXS patterns were fitted. From these fits, the spectral orientation parameters were extracted for each analyzed position on the scraps. Table 6 summarizes the corresponding values for the angular position of the diffraction maxima and their Full Width at Half Maximum (FWHM). It is worth noting that, due to the geometric relationship of diffraction, the angle measured in the two-dimensional pattern does not directly correspond to the actual fiber orientation, but is shifted by $90^{{}^{\circ}}$ with respect to it, as previously detailed.

In the virgin scrap—(A1) (Figure 7a), the equatorial plane shows higher intensity, corresponding to the predominant fiber orientation at 0°, while a weaker intensity is observed at 45°, reflecting a secondary preferred orientation. No intensity is detected in the meridional plane, since the presence of a single ply prevents the detection of scattering associated with fibers oriented at 90°. The presence of a diffuse halo confirms that the polymer matrix remains amorphous and isotropic, without preferential molecular alignment.

In Figure 7b, the calendered samples (A–D—3–4) exhibit distinct 2D WAXS patterns at different positions along the strip. In the central regions (D, C, B), away from the corners (A), a clear isotropization process occurs; not only do the diffraction peaks broaden, but their relative intensity decreases compared to the isotropic background. This indicates that fibers originally aligned at 0° and ±45° have partially lost their preferential orientation. Nevertheless, angular integration shows that the predominant diffraction bands remain at 90°, suggesting that, while overall alignment decreases, some preferred directions persist.

In contrast, near the corners (A3, A4), a strong preferential orientation persists. Interestingly, the intensity of these oriented regions is even higher than in the reference sample (A1). This effect suggests that, at the edges, fibers not only maintain their alignment but may also experience a local increase in fiber volume fraction. This is likely due to the calendering rollers squeezing the resin outward, leaving the edges ‘resin-poor’ and fiber-enriched. This displacement of resin outside the scrap boundaries, driven by roller pressure and rotation, explains the high-intensity signals observed at the specimen periphery.

**Figure 8:**
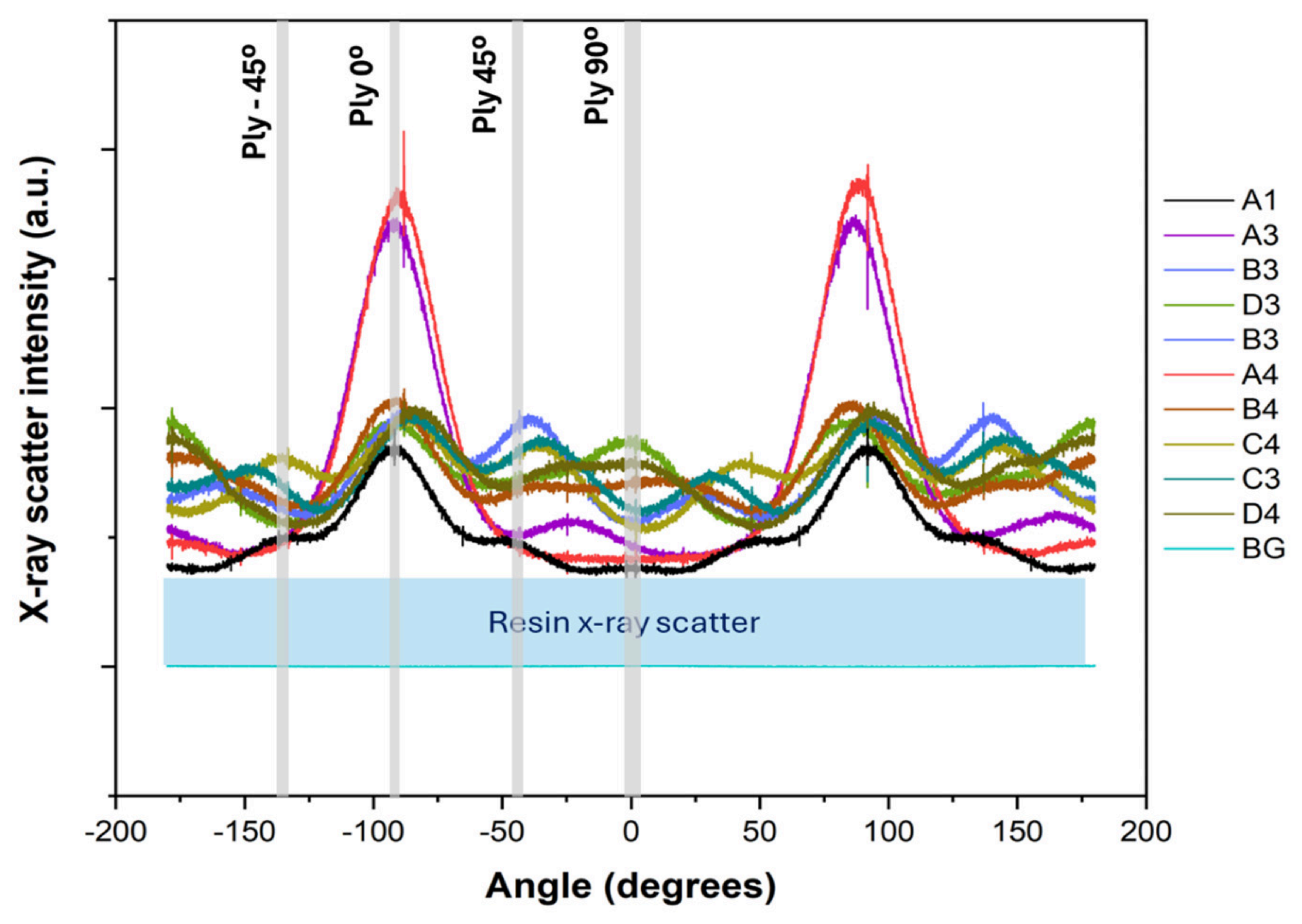
WAXS profiles of the non-calendered scraps (A1) and calendered scraps (A3–D4).

Figure 8 shows a comparative graph of the azimuthal integration of A, B, C, D, and the background (BG). It is observed that the signals exhibit an elevated baseline relative to what would be expected from the BG. This is attributed to the epoxy resin, which, due to its random orientation and amorphous phase, behaves isotropically. After calendering, in some positions (B, C, and D), the difference becomes more pronounced, likely due to the reduced fiber content, as was also observed in previous analyses.

### 3.5. Demonstrator

**Figure 9:**
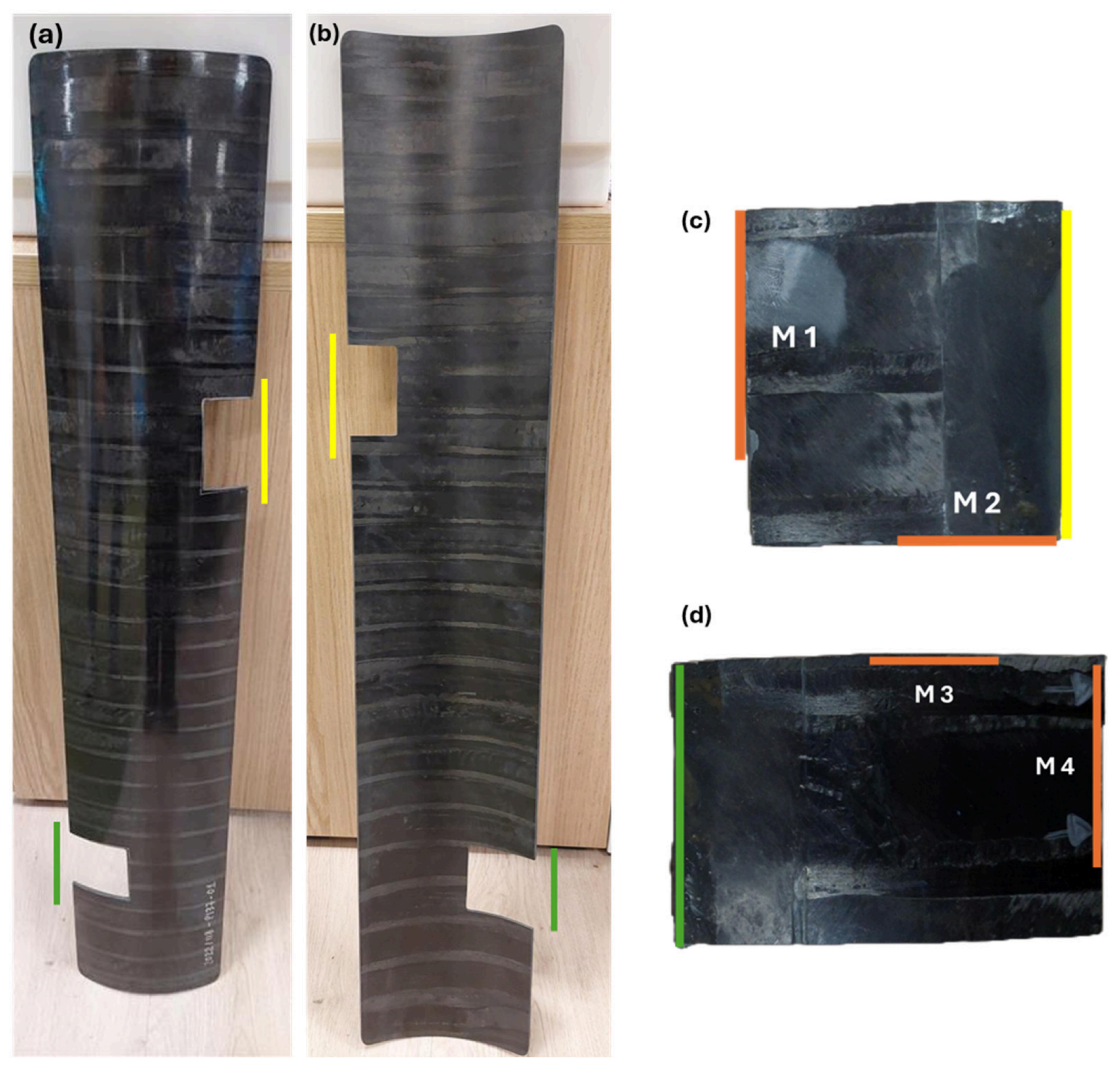
Structural demonstrator for secondary aeronautical applications. (**a**,**b**) Curved rudder skin components showing high surface quality and resin-rich finishes. (**c**,**d**) Sectioning areas (M1–M4) used to validate the industrial feasibility of the process for complex geometries. Yellow and green bars mark the outer reference edges of the extracted specimens, while orange lines denote the specific edges/surfaces evaluated under microscopic inspection.

Figure 9 shows the final panel, where no dry spots (or dry resin areas) are observed. The exterior surfaces exhibit a smooth finish with no visible roughness or marks from the strip overlaps; this is attributed to the resin-rich layers added during assembly, which ensure superior surface quality.

The outer surface (a), in contact with the female mold, presents a glossy finish, whereas the inner surface (b), in contact with the male mold, is matte, reflecting the specific surface finish of each tooling part. The final part had a thickness of 3.0 ± 0.5 mm and a weight of 1.0 ± 0.2 kg. Following fabrication, the part was machined and sectioned into two distinct sample areas (c and d) to analyze the stacking sequence, potential defects, and the presence of voids in the designated measurement locations M1 through M4.

**Figure 10:**
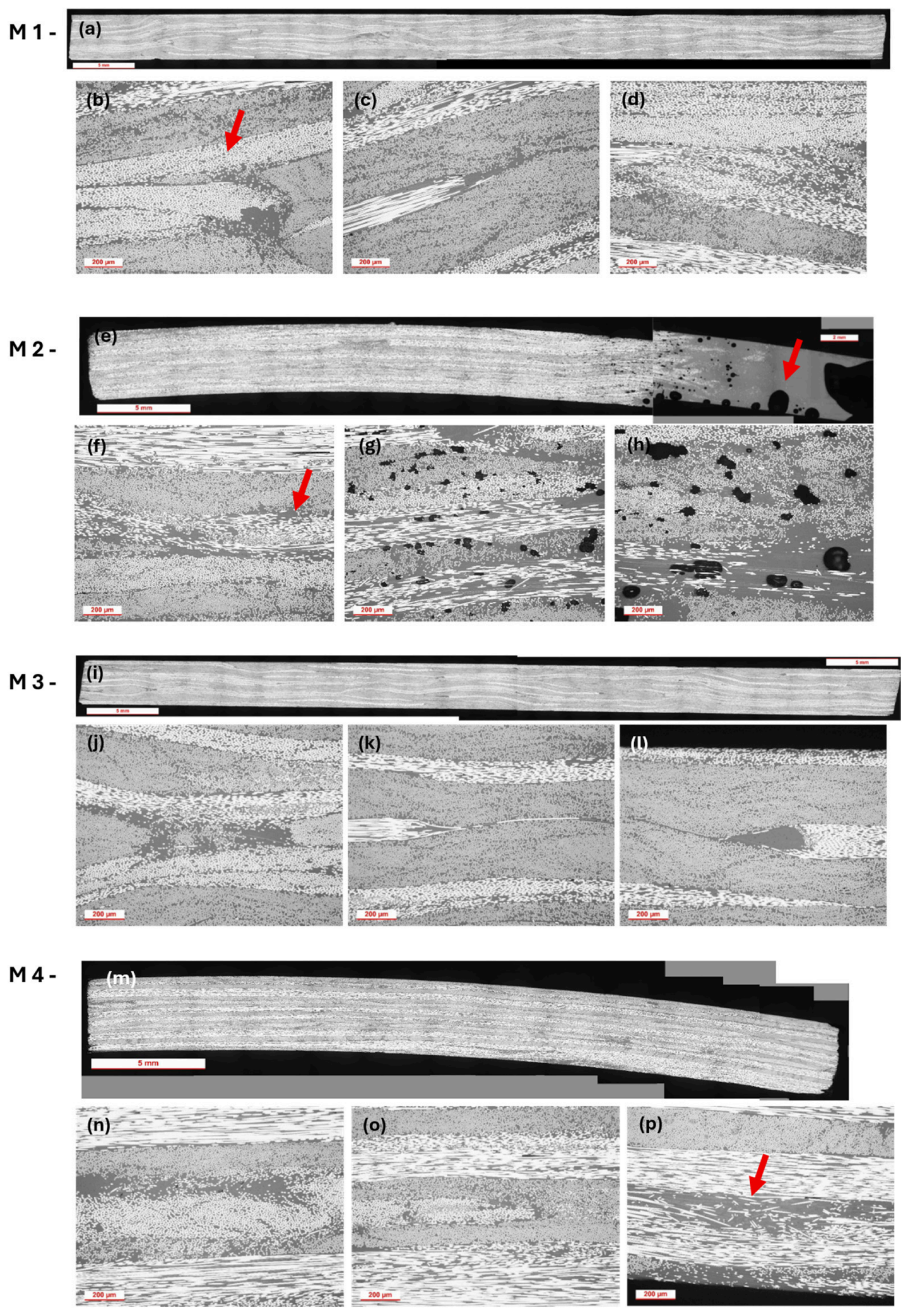
Optical micrographs of cross-sections of regions M1, M2, M3, and M4 of the composite laminate: (**a**–**d**) Region M1, showing the general cross-section (**a**) and higher-magnification views (**b**–**d**); (**e**–**h**) Region M2, showing the cross-section (**e**) and details (**f**–**h**); (**i**–**l**) Region M3, showing the cross-sectional image (**i**) and details (**j**–**l**); (**m**–**p**) Region M4, showing the cross-sectional image (**m**) and details (**n**–**p**).

The microstructural characterization of the manufactured demonstrator, as illustrated in Figure 10, reveals a high degree of consolidation and overall processing quality, characterized by a general absence of dry zones across the analyzed sections. In the first region, Sample M1 (a–d), the interface between stacked layers shows excellent wetting of resin; specifically, the red arrows in micrographs (b) and (c) highlight the overlap regions where the plies exhibit a characteristic curvature to accommodate the geometric nesting without forming resin-rich pockets. A similar structural integrity is observed in Sample M2 (e–h) within its central “useful zone” (f), where the layer nesting remains consistent. However, Sample M2 also reveals a distinct porosity distribution pattern where macro-voids and entrapped air, visible in micrographs (g) and (h), are predominantly concentrated at the lateral boundaries of the specimen. This peripheral accumulation suggests a migration or “bleeding” effect during the consolidation phase, effectively purging trapped air from the functional core toward the non-critical edges. In contrast, the analysis of Sample M4 (m–p) identifies a localized degradation of the continuous fiber architecture. Micrograph (p) shows a significant presence of fibers out of plane, indicated by the red arrow, which is attributed to the flow/misalignment during calendering.

## 4. Conclusions

One of the primary takeaways of this study is the critical role of the calendering process in redefining the material’s internal architecture. It is observed that the mechanical action of the rollers induces slight fiber disorientation, shifting the material away from its initial alignment. This shift leads to a high variability as well as a decrease in properties in 0° but produces a significant improvement in mechanical properties in the 90° orientation, a phenomenon attributed to the material’s tendency towards isotropy. While this architectural modification aims to achieve a more balanced mechanical response across different loading directions for multi-axial semi-structural applications, the high variability observed in the tensile modulus currently limits the predictability of this isotropic behavior. Further process optimization is required to reduce dispersion and ensure consistent multi-axial performance.

This phenomenon has been confirmed by X-ray diffraction (XRD) analysis performed directly on the processed strips. Heterogeneous behavior was observed in the cross-section: while the original orientation is preserved at the ends, resin loss (bleeding) occurs, and the core exhibits a quasi-random orientation. These findings validate the use of this method for the uptake of prepreg waste, enabling its application in aerospace or automotive components with moderate mechanical requirements.

Crucially, from an industrial standpoint, this methodology enables the homogenization of scrap strips, a capability that distinguishes it from alternative recycling methods. By eliminating the strict requirement of current technologies to chop, cut, or meticulously sort the waste, this process can accommodate scraps of diverse initial configurations, sizes, and geometries. Consequently, it delivers entirely homogeneous final mechanical properties, successfully overcoming the local irregularities and structural inconsistencies typical of conventional scrap processing. These findings validate the scalability of this method for the high-volume uptake of prepreg waste, enabling its viable application in aerospace or automotive components with moderate mechanical requirements.

## Figures and Tables

**Table 1:** Experimental matrix for test panel fabrication. This table details the stacking sequences and target dimensions for panels P1–P4, designed to systematically evaluate the impact of calendering-induced fiber reorientation on tensile, compression, and interlaminar shear properties.

ID	Panel Dimension (mm)	Nº of Plies per Panel	Panel Thickness (mm)	Lay-Up *	Nº of Scrap per Panel	Mechanical Test
P1	570 × 570	1	1	(0)	18	Tensile
P2	450 × 450	2	2	(0)_2_	36	ILSS Compression
P3	450 × 450	2	2	(90)_2_	36	Tensile Compression
P4	440 × 370	2	2	(0)_2_	36	-

* Lay-up refers to the overall orientation and number of layers of calendered scrap strips as they are stacked into the panel mold (e.g., (90)_2_ indicates two scrap layers stacked at a 90° orientation). Stacking sequence of the original prepreg material used for the scraps prior to calendering: [−45/+45/0_3_/90/0_3_/+45/−45].

**Table 2:** Physical properties and consolidation quality. Fiber, resin, and void content for samples. ρ: density; Wr: resin weight fraction; Wf: fiber weight fraction; Vf: fiber volume fraction; Vr: resin volume fraction; Vo: void volume fraction; S.D: standard deviation.

ID	ρ (g/cm^3^)	Wr (%)	Wf (%)	Vf (%)	Vr (%)	Vo (%) *
P1	1.58 ± 0.00	37.1 ± 2.0	62.9 ± 2.0	55.4 ± 1.85	45.0 ± 2.3	−0.4 ± 0.5
P2	1.60 ± 0.10	35.9 ± 1.2	64.1 ± 1.2	56.0 ± 1.2	43.2 ± 1.3	0.9 ± 0.5
P3	1.57 ± 0.02	33.9 ± 1.2	66.1 ± 1.2	58.2 ± 1.6	41.0 ± 1.3	0.9 ± 1.3
P4	1.58 ± 0.00	35.2 ± 0.6	64.8 ± 0.6	57.2 ± 0.7	42.7 ± 0.7	0.1 ± 0.2
Reference	1.58 ± 0.00	33.7 ± 0.2	66.3 ± 0.2	58.6 ± 0.2	41.0 ± 0.3	0.3 ± 0.3

* Negative values do not have physical sense and can be interpreted as 0.0%.

**Table 3:** Longitudinal (0°) and transverse (90°) tensile properties of reference and calendered panel, compressive strength (σC) and modulus (Ec).

Sample	Tensile Tests at 0°		Tensile Tests at 90°	
	σ_T_ (MPa)	E_T_ (GPa)	σ_T_ (MPa)	E_T_ (GPa)
Reference	1177 ± 29	74 ± 4	81 ± 5	10 ± 1
Calendered	389 ± 197	72 ± 25	87 ± 5	28 ± 1

**Table 4:** Compression tests of referenced and calendered material, compressive strength (σ_C_) and modulus (Ec).

Sample	Compression Tests at 0°		Compression Tests at 90°	
	σ_C_ (MPa)	E_C_ (GPa)	σ_C_ (MPa)	E_C_ (GPa)
Reference	1531 ± 95	125 ± 22	200 ± 10	10 ± 2
Calendered	502 ± 158	59 ± 14	243 ± 13	38 ± 4

**Table 5:** Interlaminar shear strength (ILSS) and load-bearing capacity.

Sample	Peak Load (N)	τ (MPa)
Reference	1735 ± 10	60 ± 2
Calendered	2087 ± 273	58 ± 8

**Table 6:** Spectral orientation parameters for each point on the scraps. Note that the angle does not correspond to the fiber orientation.

**A1**	**Angle (**°**)**	0	45	90	135
	**FWHM**	13.24	30.36	35.00	31.00
**A3**	**Angle (**°**)**	86.5	164.8		
	**FWHM**	37.2	51.7		
**A4**	**Angle (**°**)**	88.5			
	**FWHM**	37.3			
**B3**	**Angle (**°**)**	28.0	92.24	140.1	
	**FWHM**	21.8	33.5	37.6	
**B4**	**Angle (**°**)**	−32.2	13.9	85.1	
	**FWHM**	43.0	40.75	33.2	
**C3**	**Angle (**°**)**	31.4	93.1	147.1	
	**FWHM**	26.7	31.3	51.9	
**C4**	**Angle (**°**)**	43.5	90.0	143.9	
	**FWHM**	36.4	36.25	47.6	
**D3**	**Angle (**°**)**	−93.1	−37.0	2.8	85.8
	**FWHM**	38.1	58.8	36.9	44.1
**D4**	**Angle (**°**)**	−83.9	−27.8	8.8	96.2
	**FWHM**	40.4	42.2	37.8	44.7

## Data Availability

The original contributions presented in this study are included in the article material, and further inquiries can be directed to the corresponding authors.

## Abbreviations

The following abbreviations are used in this manuscript:

TLA	Three letter acronyms
CFRP	Carbon Fiber Reinforced Polymer
LCA	Life Cycle Assessment
ILSS	Interlaminar shear strength
DMA	Dynamic Mechanical Analysis
Tg	Glass transition temperature
WAXS	Wide-angle X-ray scattering
XRD	X-ray diffraction
Vf	Fiber volume fraction
Vr	Resin volume fraction
Vo	Void volume fraction
Wf	Fiber weight fraction
Wr	Resin weight fraction
ρ	Density
E	Elastic modulus
σ (sigma)	Strength
S.D.	Standard deviation
T0	Tensile test at 0° (longitudinal)
T90	Tensile test at 90° (transverse)
C0	Compression test at 0°
C90	Compression test at 90°
